# *Hydrilla verticillata*–Sulfur-Based Heterotrophic and Autotrophic Denitrification Process for Nitrate-Rich Agricultural Runoff Treatment

**DOI:** 10.3390/ijerph17051574

**Published:** 2020-02-29

**Authors:** Qianyu Hang, Haiyan Wang, Zan He, Weiyang Dong, Zhaosheng Chu, Yu Ling, Guokai Yan, Yang Chang, Congyu Li

**Affiliations:** 1 State Key Laboratory of Environmental Criteria and Risk Assessment, Chinese Research Academy of Environmental Sciences, No. 8 Da Yang Fang, Anwai, Chaoyang District, Beijing 100012, China; qhang@ncsu.edu (Q.H.); hezan1203@163.com (Z.H.); docreat@163.com (W.D.); lingyu18@mails.ucas.ac.cn (Y.L.); yangk@craes.org.cn (G.Y.); cy1100@126.com (Y.C.); licongyu1996@163.com (C.L.); 2 Engineering Center for Environmental Pollution Control, Chinese Research Academy of Environmental Sciences, Beijing 100012, China; 3 Beijing China’s Sustainable Development Water Purification Material Technology Co., Beijing 100012, China; 4 National Engineering Laboratory for Lake Pollution Control and Ecological Restoration, Chinese Research Academy of Environmental Sciences, No. 8 Da Yang Fang, Anwai, Chaoyang District, Beijing 100012, China

**Keywords:** *Hydrilla verticillata*, carbon source, heterotrophic denitrification, sulfur autotrophic denitrification, agricultural runoff, constructed wetland mesocosms

## Abstract

*Hydrilla verticillata*–sulfur-based heterotrophic and autotrophic denitrification (HSHAD) process was developed in free water surface constructed wetland mesocosms for the treatment of nitrate-rich agricultural runoff with low chemical oxygen demand/total nitrogen (C/N) ratio, whose feasibility and mechanism were extensively studied and compared with those of *H. verticillata* heterotrophic denitrification (HHD) mesocosms through a 273-day operation. The results showed that the heterotrophic and autotrophic denitrification can be combined successfully in HSHAD mesocosms, and achieve satisfactory nitrate removal performance. The average NO_3_^−^-N removal efficiency and denitrification rate of HSHAD were 94.4% and 1.3 g NO_3_^−^-N m^−3^·d^−1^ in steady phase II (7–118 d). Most nitrate was reduced by heterotrophic denitrification with sufficient organic carbon in phase I (0–6 d) and II, i.e., the C/N ratio exceeded 4.0, and no significant difference of nitrate removal capacity was observed between HSHAD and HHD mesocosms. During phase III (119–273 d), sulfur autotrophic denitrification gradually dominated the HSHAD process with the C/N ratio less than 4.0, and HSHAD mesocosms obtained higher NO_3_^−^-N removal efficiency and denitrification rate (79.1% and 1.1 g NO_3_^−^-N m^−3^·d^−1^) than HHD mesocosms (65.3% and 1.0 g NO_3_^−^-N m^−3^·d^−1^). As a whole, HSHAD mesocosms removed 58.8 mg NO_3_^−^-N more than HHD mesocosms. pH fluctuated between 6.9–9.0 without any pH buffer. In general, HSHAD mesocosms were more stable and efficient than HHD mesocosms for NO_3_^−^-N removal from agricultural runoff during long-term operation. The *denitrificans* containing *narG* (1.67 × 10^8^ ± 1.28 × 10^7^ copies g^−1^ mixture-soil^−1^), *nirS* (8.25 × 10^7^ ± 8.95 × 10^6^ copies g^−1^ mixture-soil^−1^), and *nosZ* (1.56 × 10^6^ ± 1.60 × 10^5^ copies g^−1^ mixture-soil^−1^) of litter bags and bottoms in HSHAD were higher than those in HHD, which indicated that the combined heterotrophic and autotrophic denitrification can increase the abundance of *denitrificans* containing *narG, nirS*, and *nosZ*, thus leading to better denitrification performance.

## 1. Introduction

Agricultural runoff always has high nitrate and low chemical oxygen demand (COD)/total nitrogen (TN) (C/N) ratio. Increased nitrate availability in agricultural runoff may lead to serious eutrophication and deterioration of public health in many cases [1,2,3]. Among the available approaches, the free water surface constructed wetland has recently been identified as an effective and ecologically sustainable technology for the treatment of agricultural runoff [4,5].

Biological denitrification, occurring either heterotrophically or autotrophically, accounts for 60–90% of the total nitrate reduction [6,7,8]. Among popular electron donors for autotrophic denitrification, sulfur has competitive characteristics of cost-effectiveness, nontoxicity, water-insolubility, and wide availability [9,10]. In the heterotrophic denitrification process, some chemical organics such as methanol and glucose are widely used as additional carbon sources for the treatment of nitrate-contaminated wastewater with a low C/N ratio [11]. However, such methods are costly due to the continuous organics dosage [12]. What is more, the residual chemical organics added need to be further treated. To substitute the chemical organics and reduce costs, various plant biomass has been proved to be feasible for the improvement of heterotrophic denitrification efficiency [13,14,15,16].

Previous studies have obtained favorable denitrification performance by applying combined heterotrophic and autotrophic denitrification (HAD) process in the treatment of drinking water [17,18,19], hydroponic wastewater [20], groundwater [21,22], simulated wastewater [23,24,25,26,27,28,29,30,31], etc. A woodchip-sulfur combined mixotrophic process was recently developed to eliminate nitrate from groundwater [31], whose denitrification performance was better than that of the sulfur-based autotrophic denitrification systems. However, it was only operated in serum bottles for less than two weeks with limited experimental data. Another study discussed the effects of coexistent ions including Na^+^, Cl^−^, HCO_3_^−^, Ca^2+^, Cu^2+^, Fe^2+^, Fe^3+^, HCO_3_^−^, and SO_4_^2−^ on nitrate removal by combined sawdust-based heterotrophic and Fe-based autotrophic denitrification processes [23]. Further investigation is needed to prove that HAD process works better than individual heterotrophic denitrification for the improvement of nitrate removal capacity during long-term operation.

Dissolved organic matter (DOM), which is one of the important components in organic compounds of plant biomass, is a key influencing factor for heterotrophic denitrification performance [32]. Recent studies have demonstrated that parallel factor analysis (PARAFAC) could decompose full excitation-emission matrix (EEM) fluorescence into different independent fluorescent groups [33], and it is a valuable tool for the characterization and quantification of DOM fluorescence changes.

Based on the studies mentioned above, the *Hydrilla verticillata* (*H. verticillata*)—sulfur-based heterotrophic and autotrophic denitrification (HSHAD) process was put forward. *H. verticillata* is a fresh-water aquatic plant used as the carbon source. This paper aims to verify the feasibility and mechanism of the HSHAD process for the treatment of high nitrate and low C/N ratio agricultural runoff in free water surface constructed wetland mesocosms during long-term operation. Characteristics of *H. verticillata* decomposition and denitrification performance in the HSHAD mesocosms were also extensively studied in long-term operation and compared with those in *H. verticillata*-based heterotrophic denitrification (HHD) mesocosms. DOM changes were also analyzed to elaborate the HSHAD mechanism.

## 2. Materials and Methods

### 2.1. Source of Material and Pretreatment

Lake Erhai is a typical plateau lake in Dali city, Yunnan Province, Southwest China. *H. verticillata* and soil samples were collected from the water near the shore and the paddy-field along the littoral zone of Lake Erhai (25°36′–25°58′ N, 100°06′–100°18′ E), respectively, in August 2014. *H. verticillata* samples were cleaned, cut into 1 to 2 cm pieces and oven-dried at 40 °C to constant weight. Soil samples were air-dried to constant weight, milled and screened through 100-mesh sieve. The granular sulfur (1–2 cm, >99.9%) and pebbles (SiO_2_, 1–2 cm) were bought from local companies.

### 2.2. Mesocosm Set-Up and Operation

Six 5 L laboratory-scale, free water surface constructed wetland mesocosms were made using plastic buckets (0.17 m bottom inner diameter, 0.23 m upper inner diameter, 0.21 m height) and fed with 2.0 L synthetic agricultural runoff. Pretreated soil from Lake Erhai shore and paddy-field were mixed homogeneously at a 1:1 mass ratio, and then added into the mesocosms. The litter bag technique could carry out the decomposition and denitrification experiments [34,35,36]. A litter bag (0.16 m length, 0.12 m width) loaded with different media (Table 1) was placed on the bottom soil (Table 1) surface in each mesocosm, and the litter bag was made of nylon with approximately 1.0 mm mesh. Two HSHAD mesocosms (packed with *H. verticillata* and sulfur in the litter bag), two HHD mesocosms (packed with only *H. verticillata* in the litter bag), and two control mesocosms (packed with only soil and gravels in the litter bag) (Table 1) were used. The schematic diagram of the mesocosm was published in a previous study [26].

Based upon one-year monitored data of the agricultural runoff in the Lake Erhai area, the ingredients of the simulated agricultural runoff were 0.5 mg L^−1^ NH_4_^+^-N, 7.4 mg L^−1^ NO_3_^−^-N, 1.2 mg L^−1^ NO_2_^−^-N, 9.1 mg L^−1^ TN, 1.2 mg L^−1^ TP, and 3.2 mg L^−1^ COD, which were achieved by the addition of 1.457 g NaNO_3_, 0.057 g NaNO_2_, 0.023 g NH_4_Cl, 0.241 g KH_2_PO_4_, and 0.419 g glucose into tap water. The pH was 8.3. The experiment was conducted in static mode by intermittent dosage of NaNO_3_ supporting *denitrificans* growth at constant NO_3_^−^-N concentration (7.4 mg L^−^^1^) for 273 days, i.e., from 14 January to 14 October 2015. The temperature was kept at 19 to 25 °C using air conditioner. Since the nitrate concentration in the control mesocosms was too high (126.1 mg L^−^^1^), the water column of all mesocosms was replaced by the simulated agricultural runoff on the 204th day.

### 2.3. Sampling and Analytical Procedure

To avoid agitation of the bottom soil, water was sampled from the top using a 50 mL syringe. Meanwhile, the same volume of deionized water was injected into the mesocosms for compensation. At the beginning and end of the experiments, cellulose, hemicellulose, and lignin contents of *H. verticillata* were quantified according to Van Soest’s method [7]. Organic carbon was analyzed by the potassium dichromate oxidation–external heating method (LY/T 1237-1999) [37]. TN was determined by automatic azotometer (UK152 Distillation and Titration Unit, Velp Co., Milano, Italy). TP was determined by inductively coupled plasma–atomic emission spectrometry (IRIS Intrepid II XSP, Thermo, Waltham, MA, USA). Before analysis, *H. verticillata* pieces were cleaned, oven-dried at 40 °C to constant weight, and then milled and screened through 60-mesh sieve.

NH_4_^+^-N and TP were determined by standard methods [38]. NO_3_^−^-N and NO_2_^−^-N were analyzed by ion chromatography (DIONEX ICS-1000, Dionex Inc., Sunnyvale, CA, USA) after 0.45 µm syringe tip-filter filtration (SCAA-201). COD was measured with a COD rapid testing instrument (model TCL-12, Hebei Chengde Huatong Environmental Instrument Ltd. Co., Chengde, China). pH was analyzed with S-25 pH Analyzer (Shanghai Precision and Scientific Instrument Ltd. Co., Shanghai, China).

DOM in water samples was characterized by three-dimensional EEM fluorescence spectrophotometer (Hitachi F-7000 Fluorescence Spectrophotometer, Japan) after glass microfiber filtration (Whatman GF/C glass microfiber filters). A xenon excitation source was used for the spectrometer. Both the excitation (Ex) and emission (Em) were 200–500 nm with 5 nm bandwidth, and the scanning speed was 12,000 nm/min. Milli-Q water was utilized as blank control. Fluorescence spectroscopy coupled with parallel factor analysis (PARAFAC) was applied in DOM fluorescence characterization. 

### 2.4. DNA Extraction and Q-PCR 

Mixed soil from the bucket bottom, and mixture (gravel/*H. verticillata* pieces/mixed soil) from litter bags were collected on the 273th day for *denitrificans* genes analysis using quantitative polymerase chain reaction (q-PCR) method. 

Before deoxyribonucleic acid (DNA) extraction, the mixture from litter bags was shaken at 200 r min^-1^ for 1 h in sterile glass bottles in order to detach the biofilm into a liquid phase. Total genomic DNA was extracted using the E.Z.N.A^®^ Soil DNA Kit (OMEGA bio-tek, Doraville, GA, USA), and was subject to q-PCR assays targeting the *narG*, *nirS,* and *nosZ* genes.

Primers were selected for the amplification of *narG* (forward primer, 1960 m2f: TA(CT)GT(GC)GGGCAGGA(AG)AAA; reverse primer, 2050 m2r: CGTAGAAGAAGCTGGTGCTGTT) [39], *nirS* (forward primer, cd3af: GTSAACGTSAAGGARACSGG; reverse primer, R3cd: GASTTCGGRTGSGTCTTGA) [40], and *nosZ* (forward primer, F:CG(C/T)TGTTC(A/C)TCGACAGCCAG; reverse primer, 1622R: CG(G/C)ACCTT(G/C)TTGCC(C/G)T(T/C)GCG) [41]. The q-PCR assays were performed on ABI 7500 fast real time PCR platform (Life Technologies, Carlsbad, CA, USA) using SYBR-green based detection. Q-PCR was conducted using a two-step amplification procedure under following conditions: 95 °C for 10 min, 40 cycles of 95 °C for 15 s, 60 °C for 1 min. A 10-fold dilution series of standard DNA was used to obtain the standard curve, which had 0.9625–0.9992 R^2^ values, and the amplification efficiency ranged from 81% to 89%. The specificity of each PCR assay was confirmed by both melting curve analysis and agarose gel electrophoresis. All the measurements were performed in triplicate.

### 2.5. Data Analysis

The volumetric denitrification rate (Equation (1)), total inorganic nitrogen (TIN) concentration (Equation (2)), and nitrate removal efficiency (Equation (3)) were calculated.

(1) $$Volumetric\;denitrification\;rate\;\left(g\;{NO_{3}}^{-}\text{-}N/m^{3}\cdot d\right)=\;\frac{∆{NO_{3}}^{-}\text{-}N\times V}{V\times ∆t}$$

(2) $$TIN=\;{NO_{3}}^{-}\text{-}N+\;{NO_{2}}^{-}\text{-}N+N{H_{4}}^{+}\text{-}N$$

∆*NO_3_^−^*-*N* (mg L*^−^*^1^), the NO_3_*^−^*-N concentration difference between any two sampling time 

*V* (L), the effective mesocosm volume 

∆*t* (d), the sampling time interval 

*NO_3_^−^*-*N* (mg L*^−^*^1^), the NO_3_*^−^*-N concentration

*NO_2_^−^*-*N* (mg L*^−^*^1^), the NO_2_*^−^*-N concentration

*NH_4_^+^*-*N* (mg L*^−^*^1^), the NH_4_^+^-N concentration

(3) $${NO_{3}}^{-}\text{-}N\;removal\;efficiency\;\left(\%\right)=\frac{{\left[{NO_{3}}^{-}\text{-}N\right]}_{na}-{\left[{NO_{3}}^{-}\text{-}N\right]}_{\left(n+1\right)b}}{{\left[{NO_{3}}^{-}\text{-}N\right]}_{na}}$$

*[NO_3_^−^*-*N]_na_* (mg L*^−^*^1^), the NO_3_*^−^*-N concentration after the number *n* time of NaNO_3_ dosing 

*[NO_3_^−^*-*N]_(n+1)b_* (mg L*^−^*^1^), the NO_3_*^−^*-N concentration before the number (*n* + 1) time of NaNO_3_ dosing

The statistical analysis was carried out using SPSS software (version 23.0, Chicago, IL, USA). The paired-sample *t*-test was used to evaluate the differences between the two mesocosms.

## 3. Results and Discussion

### 3.1. Physical-Chemical Component Variation of H. verticillata 

Cellulose, hemicellulose, and lignin are the main components of *H. verticillata* (Table 2) [42]. Cellulose and hemicellulose are macromolecules from different sugars, whereas lignin is an aromatic polymer synthesized from phenylpropanoid precursors. After 273 days’ decomposition, cellulose and hemicellulose of *H. verticillata* (0.17 and 0.23 g g*^−^*^1^ biomass*^−^*^1^) decreased to 0.09 and 0.04 g g*^−^*^1^ biomass^–1^ in HSHAD mesocosms and 0.15 and 0.03 g g*^−^*^1^ biomass*^−^*^1^ in HHD mesocosms, which indicated that sulfur does not have adverse effect on biomass decomposition. Lignin increased from 0.05 g g*^−^*^1^ biomass*^−^*^1^ to 0.22 g g*^−^*^1^ biomass*^−^*^1^ (HSHAD) and 0.15 g g*^−^*^1^ biomass*^−^*^1^ (HHD), respectively, at the same time (Table 2). 

Plant biomass decomposition converts complex organic molecules to simple organic constituents as a result of following processes: (1) physical leaching and fragmentation, (2) extracellular enzyme hydrolysis, (3) aerobic and anaerobic catabolic activities of heterotrophic microorganisms [43]. The high microbial cellulose and hemicellulose utilization (Table 2) indicated that they were likely the main carbon source for *H. verticillata* heterotrophic denitrification in the HSHAD and HHD mesocosms. Cellulose in crystalline form is less susceptible to enzymatic degradation than that in nonorganized form [44]. Crystalline cellulose accounts for a large portion of the total cellulose, which might be responsible for the large portion of the cellulose remaining in *H. verticillata* (Table 2). Hemicellulose is a complex carbohydrate polymer and more hydrolysable than cellulose [45,46], which explains the reason why most of the hemicellulose was decomposed at the end of the experiment. Lignin is the recalcitrant fraction of the plant biomass, whose microbial utilization requires initial oxygenation [43]. Therefore, the increase of lignin resulted from the nonenzymatic lignin formation and its low degree of microbial utilization [45,46,47,48]. Pérez et al. [46] confirmed that the degradation of lignin was difficult due to its structural complexity, insolubility, and high molecular weight. Moreover, 50% N and 100% P were released from *H. verticillata* in both the HSHAD and HHD mesocosms during the 273 days of operation.

### 3.2. Principles of the HSHAD Process 

Assuming the main component of the cellulose and hemicellulose in *H. verticillata* is C_6_H_10_O_5_, the stoichiometric equations of *H. verticillate* heterotrophic and sulfur autotrophic denitrification in the HSHAD process can be listed as Equations (4) and (5), respectively:

(4) $$5C_{6}H_{10}O_{5}\;+24{NO_{3}}^{-}\;+24H^{+}\;\to 12N_{2}+30CO_{2}+37H_{2}O\;\;\;$$

(5) $$55S^{0}\;+\;50{NO_{3}}^{-}\;+\;38H_{2}O+20CO_{2}+4N{H_{4}}^{+}\;\to 4C_{5}H_{7}O_{2}N+55S{O_{4}}^{2-}\;+\;25N_{2}+64H^{+}\;\;\;\;\;\;\;\;\;\;\;\;\;\;\;\;\;\;\;\;\;\;\;\;\;\;$$

Two aspects should be noted for the HSHAD process. The first aspect is its ability to keep pH balanced without a pH buffer. According to Equations (4) and (5), 1.0 mol H^+^ will be consumed when 1.0 mol NO_3_^−^-N is denitrified by *H. verticillata* heterotrophic denitrification (*H. verticillate* pieces as carbon source), and 1.0 mol H^+^ will be provided when 0.78 mol NO_3_^−^-N is reduced to N_2_ by sulfur autotrophic denitrification. If the mole ratio of nitrate reduction by *H. verticillata* heterotrophic denitrification to that by sulfur autotrophic denitrification is 1:0.78, the H^+^ generated by sulfur autotrophic denitrification can be consumed simultaneously by *H. verticillata* heterotrophic denitrification. Thus, a pH adjustment is not needed. *H. verticillata* requirement for 1.0 mol NO_3_^−^-N reduction and sulfur requirement for 0.78 mol NO_3_^−^-N reduction are then determined. Cellulose and hemicellulose of *H. verticillata* are 0.17 and 0.23 g/g·raw pieces, which account for 40.1% of the pieces’ weight (Table 2), i.e., the C_6_H_10_O_5_ component accounts for 40.1%, so the stoichiometric mass ratio of *H. verticillata* pieces to sulfur for H^+^ balance should be 3.1. Since some organic carbons (crystalline cellulose, lignin, etc.) are difficult to decompose and utilize [13], the 5.3 mass ratio of *H. verticillata* to sulfur was applied in this study.

The second aspect relates to the enhancement of denitrification performance. Theoretically, the synergistic improvement of the denitrification performance can be achieved if both the sulfur autotrophic *denitrificans* and *H. verticillata* heterotrophic *denitrificans* grow well in the HSHAD system.

### 3.3. Mesocosm Performance

#### 3.3.1. Carbon Availability

**Figure 1:**
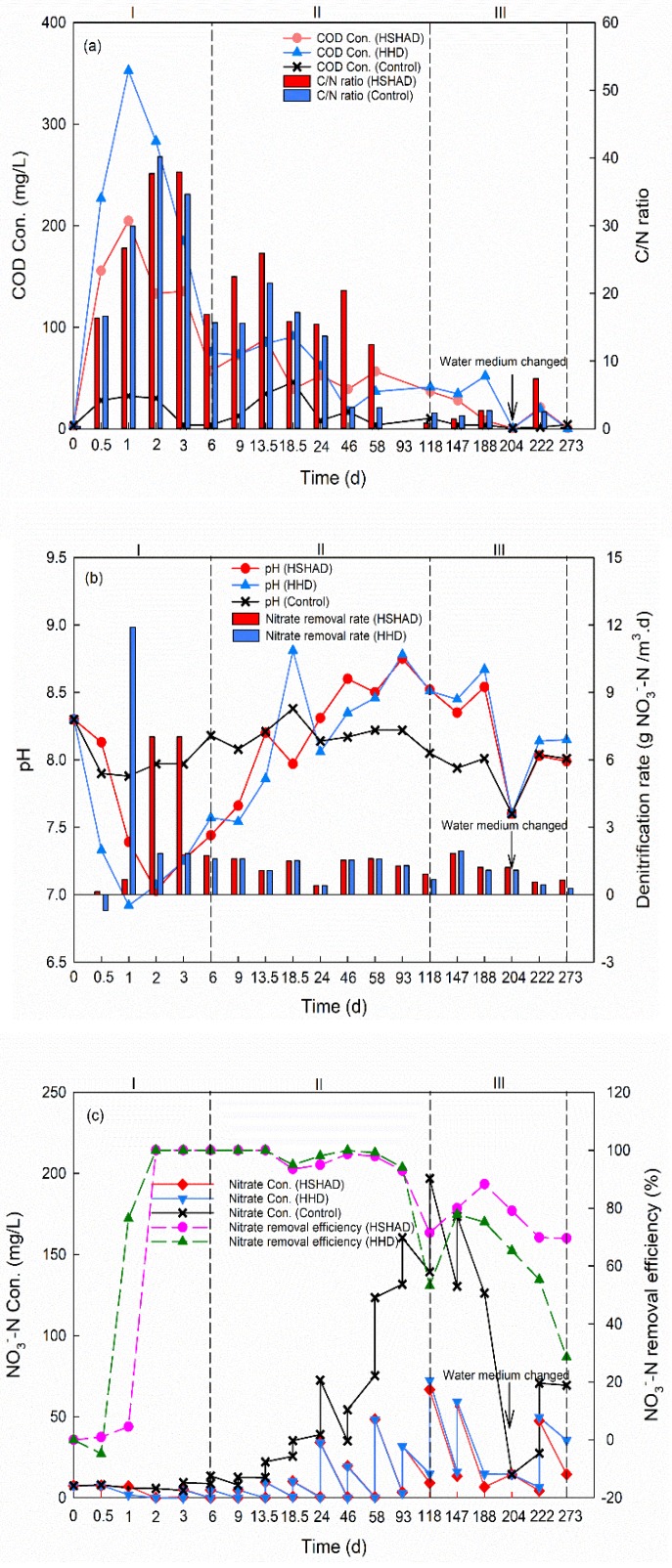
(**a**) Comparison of COD and C/N ratio profile within each mesocosm; (**b**) Comparison of pH and denitrification rate profile within each mesocosm, *p* > 0.05; (**c**) Comparison of NO_3_^−^-N concentrations and removal efficiency profile within each mesocosm.

As depicted in Figure 1a, COD in the control is low and relatively stable. Ingersoll and Baker [49] suggested that the optimum C/N ratio for plant biomass heterotrophic denitrification was 4.0–5.0. HSHAD and HHD were operated in three phases based on the C/N ratio and its fluctuation. Phase I was the short-term physical leaching period (0–6 d, C/N ratio > 4.0), during which the COD rose quickly to the maximum values (i.e., 204.7 and 352.6 mg L^−1^ for HSHAD and HHD, respectively) in 1 day (Figure 1a). The rapid COD increase resulted from the fast eluviation of water-soluble substances (i.e., organic acid, protein, and minerals) into water. The dynamic equilibrium between organic carbon release and consumption can be attained on the first day. Then COD gradually decreased to 56.9 and 74.8 mg L^−1^ in the HSHAD and HHD mesocosms on the sixth day. Phase II (7–118 d, C/N ratio > 4.0) was the long-term steady biological decay period, during which COD was released continuously at lower and steady rate from *H. verticillata* pieces to support the growth of the heterotrophic *denitrificans* [47,50]. In phase I and II, C/N ratios in HSHAD and HHD were both higher than 4.0, which meant sufficient organic carbon for heterotrophic denitrification. Phase III (119–273 d, C/N ratio < 4.0) was the COD reduction period, i.e., COD decreased gradually, which suggested that the carbon release speed was lower than its consumption speed. The average C/N ratios in HSHAD and HHD mesocosms were 2.4 and 1.4 in phase III, which meant insufficient organic carbon for heterotrophic denitrification.

The COD variation curve of HSHAD was similar to that of HHD (*p* > 0.05), indicating that there was no significant difference of organic carbon dynamics between HSHAD and HHD mesocosms.

#### 3.3.2. DOM Analysis

Four main fluorescence peaks of EEM fluorescence spectra were identified for water samples from different phases (Table 3). EEM spectral regions were related with different organic functional groups in DOM [51]. Peak C1 was located at 250–280/325–370 nm excitation/emission wavelengths (Ex/Em), which was considered as soluble microbial byproduct-like substances [51,52]. Peak C2, which was identified by 225–230/320–340 nm Ex/Em, was associated with the aromatic amino acid tryptophan, i.e., protein-like substances [51,53,54]. Peak C3 (260–360/410–460 nm Ex/Em) and Peak C4 (230–250/460–480 nm Ex/Em) were described as humic acid-like and fulvic acid-like substances, respectively [51,52,55,56]. 

**Figure 2:**
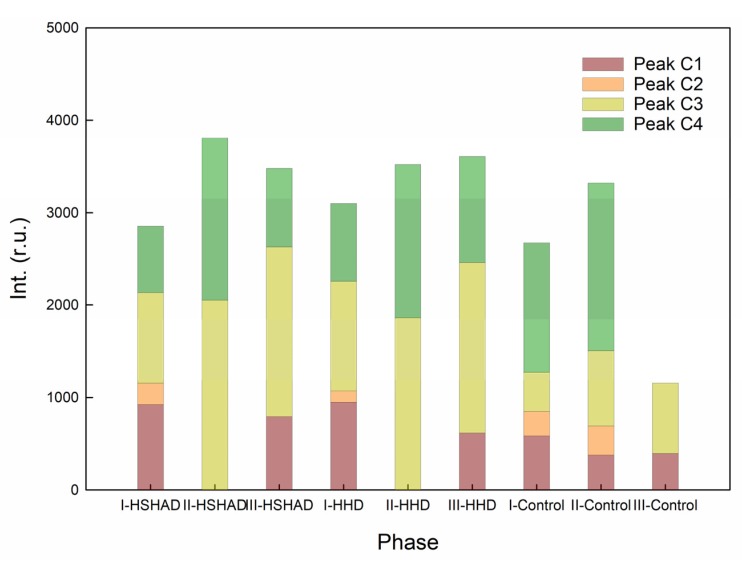
Compositional proportions of different peak intensities for HSHAD and HHD mesocosms during the operation.

Figure 2 and Table 3 illustrate the compositional proportion of each peak intensity in different phases (119–203 d of Phase III), and obvious shifts of peak intensity were observed. From phase I to phase II, rapid decrease of the fluorescence intensity ratio of Peak (C1 + C2) to Peak (C3 + C4) was observed. It can be seen from Figure 2 that massive water-soluble substances (e.g., tryptophan, Peak C1) were released initially, and the fulvic acid-like (Peak C3) and humic acid-like (Peak C4) substances were produced throughout the long-term biological decay process. Fluorescence intensity of Peak (C3 + C4) were highest in phase II for all mesocosms.

In the compost, the typical mature components were characterized as humic and fulvic acid-like substances (Peak C3 and C4), while the typical immature components contained tryptophan-like substances and soluble microbial byproduct-like substances (Peak C1 and C2) [33]. The decomposition process can be characterized by the degradation of original tyrosine-like and tryptophan-like material (Peak C1 and C2) and the increase of humic and fulvic-like material (Peak C3 and C4) [33,57]. Therefore, it can be concluded from the peak alternation (Figure 2) that the soil and *H. verticillata*/sulfur (or *H. verticillata*) mixture in the experimental mesocosms were gradually matured in Phase II, which corresponded to the steady COD concentration and nitrate removal (Figure 1).

Almost no peak was found after 204 days of operation when the water medium of the mesocosms was displaced by the simulated agriculture runoff on the 204th day in phase III (data are not shown), which corresponded to the low COD concentration (around 10.0 mg/L) and denitrification rate (Figure 1), and it further confirmed that organic compound release from *H. verticillata* attained the decline period.

#### 3.3.3. Nitrogen Removal and SO_4_^2−^ Generation

NO_3_^−^-N removal performance of HSHAD and HHD is shown in Figure 1b,c. Biological denitrification processes need acclimation. In phase I, the denitrification rate rapidly increased to 7.0 g NO_3_^−^-N m^−3^·d^−1^ on the second day for HSHAD and 11.9 g NO_3_^−^-N m^−3^·d^−1^ on the first day for HHD, then it sharply dropped to 1.7 and 1.8 g NO_3_^−^-N m^−3^·d^−1^, respectively, on the sixth day (Figure 1b). During phase II, the denitrification rate remained quite stable with an average of 1.3 and 1.2 g NO_3_^−^-N m^−3^·d^−1^ for the HSHAD and HHD mesocosms. In phase III, the average denitrification rates of HSHAD and HHD gradually decreased to 1.1 and 1.0 g NO_3_^−^-N m^−3^·d^−1^ because of the absence of organic carbon. A similar trend of denitrification rate was observed for HSHAD and HHD mesocosms, but the average denitrification rate of HSHAD was higher than that of HHD in phase II and III. The denitrification rate of HSHAD gradually increased from the 222th day, but that of HHD still decreased. On the 273th day, the denitrification rate of HSHAD mesocosms (0.7 g NO_3_^−^-N m^−3^·d^−1^) was 1.3 times higher than that of HHD mesocosms (0.3 g NO_3_^−^-N m^−3^·d^−1^).

For both HSHAD and HHD mesocosms, the same maximum NO_3_^−^-N removal value (100.0%) was achieved on the second day. Then, the NO_3_^−^-N removal efficiency was maintained above 90.0% until the 118th day. No statistical differences were observed in NO_3_^−^-N removal efficiency between HSHAD and HHD mesocosms during phase I and II (*p* > 0.05) (Figure 1a), which might be due to the sufficient organic carbon released from plant biomass decomposition [17]. After 118 days of operation, organic carbon inadequacy became an obvious limiting factor for NO_3_^−^-N reduction. NO_3_^−^-N removal efficiency of HSHAD and HHD both decreased slowly. What is more, NO_3_^−^-N removal efficiency of HHD decreased faster than that of HSHAD, which demonstrated that the HSHAD process was more effective and efficient than the individual *H. verticillata* heterotrophic dentirification process when the carbon source was insufficient. So the denitrification rate of HSHAD was higher than that of HHD in phase II and III, and the NO_3_^−^-N removal efficiency of HSHAD was also higher than that of HHD after 118 days of operation. On the 273th day, NO_3_^−^-N removal efficiency of HSHAD (69.6%) was still 41.0% higher than that of HHD (28.6%), which might be caused by the combination of sulfur autotrophic denitrification.

Total NO_3_^−^-N reduction by HHD was calculated using the stoichiometric equation of the heterotrophic denitrification process (Equation (4)). Theoretically, 912.8 mg NO_3_^−^-N can be reduced by HHD if cellulose and hemicellulose reduced (Table 2) in *H. verticillata* pieces were completely utilized by heterotrophic denitrification. However, only 557.8 mg NO_3_^−^-N was eliminated within 273 days of operation, i.e., 61% of cellulose and hemicellulose reduction was used by heterotrophic denitrification, and the other 39% might be used by anaerobic and aerobic respiration rather than by heterotrophic denitrification, which was consistent with the 39% COD consumption for anaerobic and aerobic respiration reported in wetlands [47]. Total NO_3_^−^-N reduction achieved by HSHAD mesocosms (616.6 mg) was 58.8 mg greater than that by HHD mesocosms (557.8 mg), which was attributed to sulfur autotrophic denitrification.

In general, the HSHAD process was split into two major stages in this study. One stage occurred in the period 0–118 d (phase I and II), during which heterotrophic denitrification dominated at higher C/N ratio (above 4.0); the other stage spanned 119–273 d (phase III), during which sulfur autotrophic dentirification dominated at lower C/N ratio (less than 4.0), which was different from the simultaneous oxidation of sulfur (or thiosulfate) and organic matter by inoculating *denitrificans* or activated sludge into heterotrophic and autotrophic denitrification systems [20,58,59,60]. However, this phenomenon agreed well with the findings of Liu et al. [17], i.e., nitrate was primarily reduced by heterotrophic denitrification when the carbon source was adequate, and the residual was subsequently reduced by autotrophic sulfur denitrification. Also, the balanced growth of autotrophic and heterotrophic *denitrificans* was difficult to achieve without microbial inoculation probably because of the very different growth rates for both bacterial groups [61]. To a great extent, the advantage of HSHAD was its higher nitrate removal capacity and more stable denitrification performance compared with HHD during the later operation period (119–273 d).

**Figure 3:**
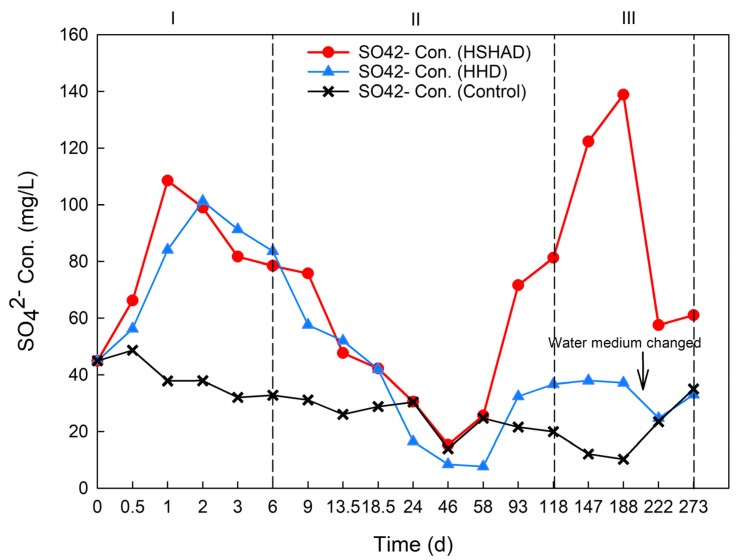
SO_4_^2−^ characteristics of each mesocosm during the experimental period.

To further demonstrate the critical function of sulfur autotrophic denitrification during the later carbon-limited period (119–273 d), SO_4_^2^^−^ concentrations for HSHAD, HHD, and control mesocosms were analyzed (Figure 3). No notable differences were observed in SO_4_^2^^−^ concentrations between the HSHAD and HHD mesocosms until the 93th day. A total of 680.2 mg more SO_4_^2^^−^ was produced in HSHAD mesocosms in comparison to HHD during the whole operation. This occurred because SO_4_^2^^−^ concentrations increased after 46 days of operation (Figure 3) with higher proportion of NO_3_^−^-N reduced by sulfur autotrophic denitrification in the HSHAD mesocosms.

In fact, corresponding to the extra 58.8 mg NO_3_^−^-N reduction in HSHAD mesocosms, 443.6 mg SO_4_^2^^−^ should be produced, based on Equation (5), which was lower than the measured value of 680.2 mg. This phenomenon suggests that SO_4_^2^^−^ generation did not completely obey the stoichiometric relationship due to sulfite and thiosulfate formation by some intermediate reaction processes [31,62]. 

Concentrations of NH_4_^+^-N, NO_2_^−^-N and TP during different phases were also analyzed. Average NO_2_^-^-N concentrations were 1.7 (2.0), 2.9 (2.8) and 1.9 (2.0) mg L^–1^ for HSHAD (HHD) mesocosms in phase I, II, and III. By comparison with the data for HSHAD, HHD, and control, fast nutrient release from *H. verticillata* at the beginning (phase I) resulted in a sharp increase in NH_4_^+^-N and TP concentrations for both HSHAD and HHD mesocosms, which was similar with former research [48,63,64,65]. However, at phase II and III, NH_4_^+^-N and TP concentrations averaged 1.1 (1.2) and 1.0 (0.9) mg/L for HSHAD (HHD) mesocosms, which demonstrated that the initial NH_4_^+^-N and TP accumulation can be successfully removed after phase I. Presumably, the effluent NH_4_^+^-N, NO_2_^−^-N, and TP can be less likely accumulated if the experiment was operated in a continuous feeding mode.

#### 3.3.4. pH Change

The pH in both HSHAD and HHD mesocosms presented a similar trend and fluctuated between 6.9 and 9.0 throughout the entire operation (Figure 1b). The pH of HSHAD mesocosms sharply decreased from 8.3 to a minimum value of 7.0 on the second day, and slowly increased to 8.8 on the 93th day, and then declined to 7.6 on the 204th day. The sharp pH decrease within two days may have been caused by aerobic respiration. In phase III, the pH of the HSHAD mesocosms was slightly lower than that of HHD, which resulted from more NO_3_^-^-N removal by sulfur autotrophic denitrification. pH had a closed relation with *denitrificans* growth, and therefore influenced denitrification performance [66]. The optimum pH for most strains of heterotrophic *denitrificans* is 7.0–8.0 [67,68], while that for sulfur autotrophic *denitrificans* is 7.7–8.6 [68]. After 46 days of operation, pH in both of HSHAD and HHD exceeded the optimum range for heterotrophic *denitrificans* growth (Figure 1b), which may have led to the decline of denitrification performance. After 9 days of operation, the pH in HSHAD was within the optimum range for autotrophic *denitrificans*. Sulfur autotrophic *denitrificans* could grow well under such pH conditions in HSHAD, which resulted in the higher denitrification capability of HSHAD than HHD in phase II and phase III. 

### 3.4. Mesocosm Denitrifying Genes

**Figure 4:**
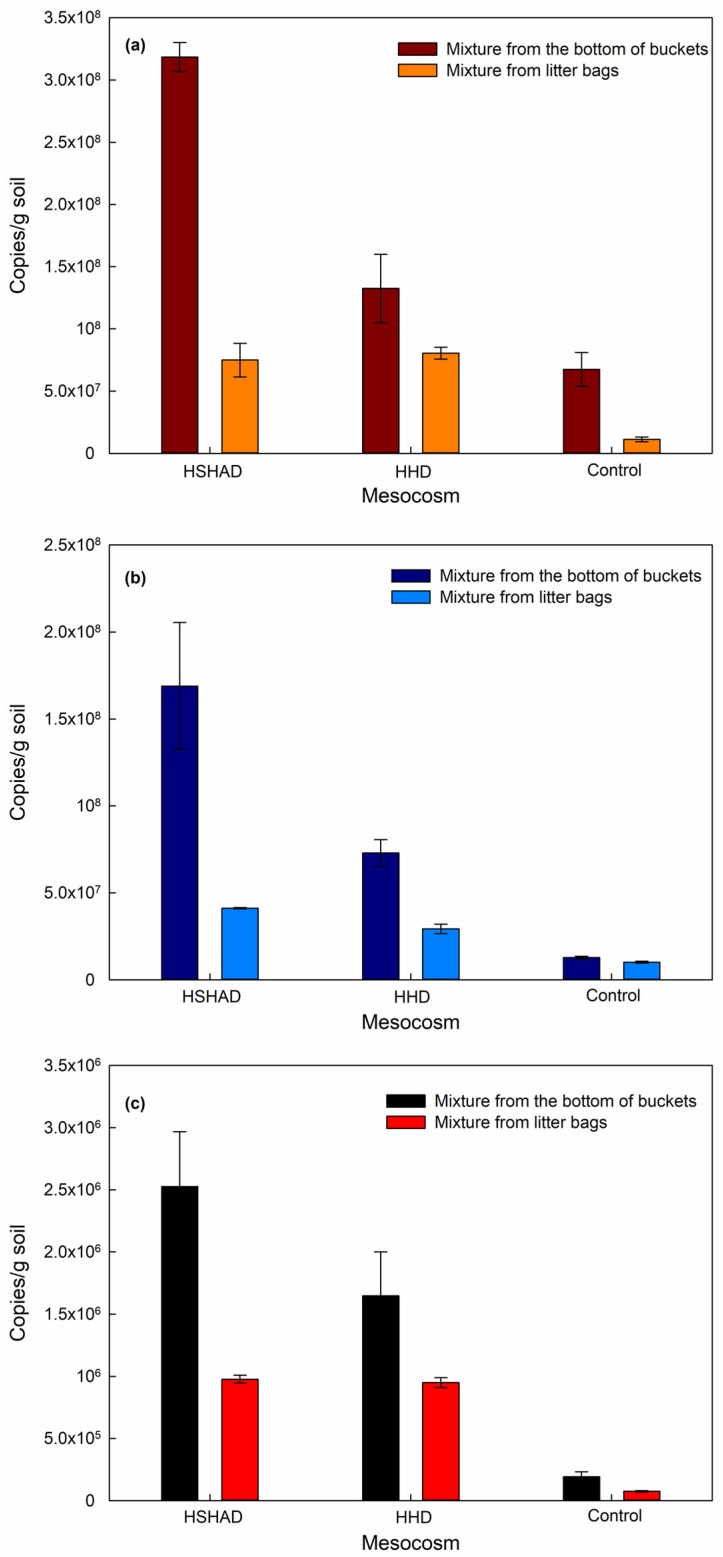
The abundance of denitrifying *narG* (**a**)*, nirS* (**b**)*,* and *nosZ* (**c**) genes in mesocosms (error bars represent standard errors of the mean value).

The total abundance of denitrifying genes (*narG*, *nirS*, and *nosZ*) in the mesocosms were calculated using their corresponding genes copies to the weight of the DNA samples, and the average abundance of denitrifying genes with standard errors in bottom soil and in mixture from litter bags for HSHAD, HHD, and control mesocosms on the 273th day are illustrated and characterized in Figure 4a–c. 

The *nirS* genes in the bottom soil and mixture from litter bags of HSHAD mesocosms were 1.7 × 10^8^ ± 3.7 × 10^7^ copies g^−1^ soil^−1^ and 4.1 × 10^7^ ± 3.4 × 10^5^ copies g^−1^ mixture^−1^, which were much higher than those of HHD mesocosms (7.3 × 10^7^ ± 7.6 × 10^6^ copies g^−1^ soil^−1^ and 2.9 × 10^7^ ± 2.7 × 10^7^ copies g^−1^ mixture^−1^) (Figure 4b). The *nosZ* genes in the bottom soil presented the same trend as *nirS* genes, and their abundance in HSHAD mesocosms (2.5 × 10^6^ ± 4.4 × 10^5^ copies g^−1^ soil^−1^) was higher than that in HHD mesocosms (1.6 × 10^6^ ± 3.5 × 10^5^ copies g^−1^ soil^−1^) (Figure 4c). Similarly, the abundance of *narG* genes (3.2 × 10^8^ ± 1.2 × 10^8^ copies g^−1^ soil^−1^) in the bottom soil of HSHAD mesocosms was greater than that of HHD mesocosms (1.3 × 10^8^ ± 2.8 × 10^7^ copies g^−1^ soil^−1^) (Figure 4a). The *narG, nirS*, and *nosZ* genes in the bottom soil and the *nirS* genes in the mixture of HSHAD mesocosms were all higher than those of HHD mesocosms, and one possible explanation was that the combined heterotrophic and autotrophic process could have improved the growth of *denitrificans* with *narG, nirS, nosZ* genes in the bottom soil and *nirS* in the mixture of HSHAD. However, the *nosZ* and *narG* genes in the mixture from the litter bags of HSHAD mesocosms were 9.8 × 10^5^ ± 3.1 × 10^4^ and 7.5 × 10^7^ ± 1.3 × 10^7^ copies g^−1^ mixture^−1^, which were similar with those of HHD mesocosms (9.5 × 10^5^ ± 3.9 × 10^4^ and 8.0 × 10^7^ ± 4.8 × 10^7^ copies g^–1^ mixture^−1^) (Figure 4a,c).

The total *denitrificans* genes content of the mesocosms was calculated using all the genes copies to the total weight of the litter bag mixture DNA sample and bottom DNA sample. The results showed that HSHAD could supply more favorable circumstances for the growth of *denitrificans* containing *narG* (1.7 × 10^8^ ± 1.3 × 10^7^ copies g^−1^ mixture-soil^−1^), *nirS* (8.2 × 10^7^ ± 8.9 × 10^6^ copies g^−1^ mixture-soil^−1^), and *nosZ* (1.6 × 10^6^ ± 1.6 × 10^5^ copies g^−1^ mixture-soil^−1^) than HHD, i.e., 1.0 × 10^8^ ± 1.0 × 10^7^, 4.9 × 10^7^ ± 4.9 × 10^6^ and 1.3 × 10^6^ ± 1.8 × 10^5^ copies g^−1^ mixture-soil^−1^, respectively (Figure 4). It can be deduced that sulfur addition might contribute to the increase of *narG*, *nirS*, and *nosZ* genes in HSHAD. So the HSHAD had better denitrification performance than HHD in phase III. The total abundance of *narG*, *nirS*, and *nosZ* genes (4.2 × 10^7^ ± 6.6 × 10^6^, 1.2 × 10^7^ ± 5.2 × 10^5^ and 1.4 × 10^5^ ± 2.1 × 10^4^ copies g^−1^ mixture-soil^−1^) in control mesocosms were much less than those in HSHAD and HHD mesocosms, which demonstrated the positive role played by the *H. verticillata* carbon sources in the growth of denitrifying bacteria.

The abundance of *nosZ* genes was lower than that of *narG* and *nirS* genes, which was similar to previous studies [69,70]. The *nosZ* genes catalyzed the reduction of N_2_O to N_2_ and enhanced the growth of bacteria containing the *nosZ* gene, thus reduced the emission of N_2_O. However, it has been reported that approximately one-third of genome denitrifying bacteria have N_2_O reductase [71].

### 3.5. Comparison of HSHAD with Other HAD Processes

The nitrate removal performance of the HSHAD process was compared to that of other HAD processes, based on literature survey, and the packed fillers and the highest removal capacity of each process are listed in Table 4. In a pilot-scale, horizontal-flow constructed wetland, only 67.0% NO_3_^−^-N removal efficiency was attained because of inadequate influent organic carbon [20,60]; however, the HSHAD mesocosms could attain the high NO_3_^−^-N removal efficiency (100%). In bioreactors, most HAD processes could achieve above 89.0% NO_3_^−^-N removal efficiency with the highest denitrification rate of 5050.0 g NO_3_^−^-N/m^3^·d (Table 4), and HSHAD mesocosms could attain the highest NO_3_^−^-N removal efficiency (100%) as the reported HAD bioreactors (89–100%). The highest denitrification rate of HSHAD (7.0 g NO_3_^−^-N m^−3^ d^−1^) was close to the double-layer permeable reactive barrier (9.5 g NO_3_^−^-N m^−3^ d^−1^) and the packed-bed bioreactor (5.0 g NO_3_^−^-N m^−3^·d^−1^) [19,21], but was lower than other reported bioreactors (5.0–5050.0 g NO_3_^−^-N m^−3^ d^−1^) [17,28,31,60,72,73,74]. Different denitrification performance probably resulted from the abundance of denitrifying bacteria, reactor configuration, type of carbon source, denitrification microenvironment, operation conditions, etc. Therefore, the optimum parameters (e.g., plant biomass species, mass ratio of plant biomass/sulfur, soil types, dosing positions of plant biomass, sulfur, etc.) should be further investigated to obtain more desirable nitrate removal performance by HSHAD in free water surface constructed wetlands.

## 4. Conclusions

To effectively treat nitrate-rich agricultural runoff with low C/N ratio, the HSHAD process in free water surface constructed wetland mesocosms was put forward, and its performance was extensively evaluated and compared with that of HHD in mesocosms through a 273-day operation.

1. Heterotrophic and autotrophic denitrification can be combined in the HSHAD process, i.e., the former process mainly dominated NO_3_^−^-N reduction during the 0–118 days of operation with 4.0 or higher C/N ratio, while the latter process dominated during 119–273 days of the operation.
2. The average NO_3_^−^-N removal efficiency and denitrification rate of HSHAD mesocosms were 94.4% and 1.3 g NO_3_^−^-N m^−3^·d^−1^ in steady phase II (7–118 d). The HSHAD process was much more efficient and stable than the HHD process in the long-term operation. At the end of the experiment, the NO_3_^−^-N removal efficiency of HSHAD mesocosms (69.6%) was 41.0% higher than that of HHD mesocosms (28.6%). The rapid increase of NH_4_^+^-N, NO_2_^−^-N, and TP concentration in the beginning did not affect the HSHAD denitrification performance, and a pH buffer was not necessary for its moderate fluctuation throughout the operation.
3. The combination of *H. verticillate* pieces heterotrophic and sulfur autotrophic denitrification led to the higher total abundance of *denitrificans* containing *narG* (1.67 × 10^8^ ± 1.28 × 10^7^ copies g^−1^ mixture-soil^–1^)*, nirS* (8.25 × 10^7^ ± 8.95 × 10^6^ copies g^−1^ mixture-soil^−1^), and *nosZ* (1.56 × 10^6^ ± 1.60 × 10^5^ copies g^−1^ mixture-soil^−1^) in the litter bags and bottoms of HSHAD mesocosms than that of HHD, which thus resulted in better denitrification performance.

## Figures and Tables

**Table 1:** Details of experimental set-up.

Mesocosm	Plant	Mass Composition in Litter Bags (g) *				Bottom Soil Layer (g) *	Simulated Wastewater (L) *
		Biomass	Sulfur	Soil	Gravel **		
HSHAD	*H. verticillata*	10.0	1.9	42.9	28.6	57.1	2.0
HHD	*H. verticillata*	10.0	0	42.9	28.6	57.1	2.0
Control	--	0	0	42.9	28.6	57.1	2.0

* The values were taken from the duplicate samples. ** The gravel was used to sink the litter bags into the surface of bottom soil layer. HSHAD (*Hydrilla verticillata*–sulfur-based heterotrophic and autotrophic denitrification, HHD (*H. verticillata* heterotrophic denitrification).

**Table 2:** Changes of the main composition at the end of the experiment (per g *H. verticillate* pieces).

Group	Type	Cellulose (g) *	Hemicellulose (g) *	Lignin (g) *	N (g) *	P (g) *
Raw	*H. verticillata*	0.17	0.23	0.05	0.02	0.01
End in HSHAD	*H. verticillata*	0.09	0.04	0.22	0.01	0
End in HHD	*H. verticillata*	0.15	0.03	0.15	0.01	0

* The values were taken from the duplicate samples.

**Table 3:** Spectral characteristics of excitation (Ex) and emission (Em) maxima of four components in each phase (Intensity (Int.)).

Mesocosm	Peak C1		Peak C2		Peak C3		Peak C4		C1+C2/C3+C4
	Ex/Em (nm)	Int. (r.u.)	Ex/Em (nm)	Int. (r.u.)	Ex/Em (nm)	Int. (r.u.)	Ex/Em (nm)	Int. (r.u.)	
HSHAD (Phase I)	280/340	1138.1	230/340	310.9	330/410,360/460	699.2	245/460	358.3	1.4
HHD (Phase I)	270/345	1340.9	225/340	326.7	330/430	1133.9	245/460	858.9	0.8
Control (Phase I)	275/325	620.0	230/320	343.9	NA ^a^	NA ^a^	240/480	1467.4	0.7
HSHAD (Phase II)	NA ^a^	NA ^a^	NA ^a^	NA ^a^	325/425,275/425	1808.5	230/470	2093.5	0
HHD (Phase II)	NA ^a^	NA ^a^	NA ^a^	NA ^a^	330/425,275/425	1659.6	230/460	3342.2	0
Control (Phase II)	275/325	403.2	225/325	328.1	325/425,260/425	552.0	230/460	2023.7	0.3
HSHAD (Phase III)	280/370	801.2	NA ^a^	NA ^a^	330/420,260/420	1834.8	245/460	848.8	0.3
HHD (Phase III)	280/370	624.4	NA ^a^	NA ^a^	330/415,260/415	1842.9	250/460	1146.3	0.2
Control (Phase III)	250/370	404.7	NA ^a^	NA ^a^	330/425,260/425	760.4	NA ^a^	NA ^a^	0.5

^a^ No data available. Values are means of each phase in mesocosms.

**Table 4:** Comparison of biological denitrification capacity among different approaches.

Denitrification Approach	Packing Material/Electron Donors	System Description	Maximum Nitrate Removal Efficiency (%)	Maximum Nitrate Denitrification Rate (g m^−3^·d^−1^)	References
HSHAD	Sulfur/*H. verticillata*/gravel/wetland and paddy soil	Free water surface constructed wetland mesocosm	100	7.0	This study
HAD	Sulfur/methanol/anaerobic sludge/sulfur autotrophic *denitrificans*	Sulfur packed bed reactor	89	5050.0 ^b^	[60]
HAD	Sulfur/methanol/aerobic and anaerobic sludge/	Sulfur particle master culture reactor	>97 ^b^	1920.0 ^b^	[28]
HAD	Sulfur/acetate/anaerobic sludge/sulfur autotrophic *denitrificans*	Sulfur particle master culture reactor	100 ^b^	98.9 ^b^	[73]
HAD	Pine bark/spongy iron/sand/gravel/heterotrophic and autotrophic *denitrificans*	Double-layer permeable reactive barrier	99 ^b^	9.5 ^b^	[21]
HAD	Sulfide/sulfide-degrading bacteria (*Pseudomonas sp. C27*)/acetate	Expanded granular sludge bed	100 ^b^	NA ^a^	[75]
HAD	Sulfur/heterotrophic and autotrophic *denitrificans*/methanol	Fluidized bed reactor	−100 ^b^	1440.0 ^b^	[17]
HAD	Cotton/zero valent iron (R4)/bacteria inoculation	Double layer column reactor	−100 ^b^	275.0 ^b^	[72]
HAD	Sulfide/acetate	Anaerobic continuous stirred tank reactor	100	−13.8 ^b^	[74]
HAD	Sulfur/limestone/methanol	Lab-scale packed-bed bioreactor	100 ^b^	5.0 ^b^	[19]
HAD	Methanol/anaerobic sludge	Intensified biofilm-electrode reactor	97	−146.0	[22]
HAD	Seed sludge/sulfur/woodchips/ /*Thiobacillus* bacteria inoculation	Serum bottle reactor	100 ^b^	−18.0 ^b^	[31]
HAD	*Thiobacillus* bacteria inoculation/sulfur/limestone	Pilot-scale horizontal flow CW	−67 ^b^	NA ^a^	[20]
HAD	Ethanol/sulfur sesquioxide/anaerobic sludge	Serum bottle reactor	100 ^b^	NA ^a^	[76]

^a^ No data available. ^b^ Estimated or calculated based on the data of the references.
